# Regulation of fear extinction versus other affective behaviors by discrete cortical scaffolding complexes associated with NR2B and PKA signaling

**DOI:** 10.1038/tp.2015.150

**Published:** 2015-10-13

**Authors:** K A Corcoran, K Leaderbrand, V Jovasevic, A L Guedea, F Kassam, J Radulovic

**Affiliations:** 1 Department of Psychiatry and Behavioral Sciences, Feinberg School of Medicine, Northwestern University, Chicago, IL, USA

## Abstract

In patients suffering from post-traumatic stress disorder (PTSD), fear evoked by trauma-related memories lasts long past the traumatic event and it is often complicated by general anxiety and depressed mood. This poses a treatment challenge, as drugs beneficial for some symptoms might exacerbate others. For example, in preclinical studies, antagonists of the NR2B subunit of *N*-methyl-d-aspartate receptors and activators of cAMP-dependent protein kinase (PKA) act as potent antidepressants and anxiolytics, but they block fear extinction. Using mice, we attempted to overcome this problem by interfering with individual NR2B and PKA signaling complexes organized by scaffolding proteins. We infused cell-permeable Tat peptides that displaced either NR2B from receptor for activated C kinase 1 (RACK1), or PKA from A-kinase anchor proteins (AKAPs) or microtubule-associated proteins (MAPs). The infusions were targeted to the retrosplenial cortex, an area involved in both fear extinction of remotely acquired memories and in mood regulation. Tat-RACK1 and Tat-AKAP enhanced fear extinction, all peptides reduced anxiety and none affected baseline depression-like behavior. However, disruption of PKA complexes distinctively interfered with the rapid antidepressant actions of the *N*-methyl-D-aspartate receptors antagonist MK-801 in that Tat-MAP2 blocked, whereas Tat-AKAP completely inverted the effect of MK-801 from antidepressant to depressant. These effects were unrelated to the MK-801-induced changes of brain-derived neurotrophic factor messenger RNA levels. Together, the findings suggest that NR2B–RACK1 complexes specifically contribute to fear extinction, and may provide a target for the treatment of PTSD. AKAP-PKA, on the other hand, appears to modulate fear extinction and antidepressant responses in opposite directions.

## Introduction

Fear extinction is based on learning that cues and situations associated with past stressful events no longer predict such outcomes, but patients with post-traumatic stress disorder (PTSD) fail to extinguish fear even decades after a traumatic event.^1^ The persistence of memory-related fear symptoms is often exacerbated by negative alterations of arousal and mood,^2, 3^ which affect up to 50% of PTSD patients.^4, 5, 6^ This causes a serious treatment problem in general, and for chronic PTSD in particular, when PTSD/depression become indistinguishable.^7^ Retrosplenial cortex (RSC), a part of midline posterior cortices (posterior cingulated cortices in primates) involved in cognitive and affective processes,^8^ is one of the brain areas showing abnormal function in patients not only with acute but also with chronic PTSD^9, 10, 11, 12, 13^ as well as major depression.^14, 15^ This suggests that mechanisms within this brain area might contribute to the fear, anxiety and mood symptoms accompanying PTSD. Recent rodent studies support this view by showing that retrieval and extinction of remote stress-related memories depend on the RSC;^16, 17^ however, evidence for a role in other behavioral phenotypes is still lacking.

There is consistent evidence from preclinical studies that activation of the NR2B subunit of *N*-methyl-d-aspartate receptor and inhibition of protein kinase (PKA) activity in different areas of fear and memory circuits are required for extinction of recently and remotely acquired fear.^17, 18, 19, 20, 21^ For context-specific memories, the circuits initially involve the hippocampus, amygdala and infralimbic cortex;^22^ however, with time these memories become hippocampus independent,^23^ and seem to be predominantly regulated by cortical mechanisms.^24^ Accordingly, extinction of remote contextual fear required upregulation of NR2B activity and downregulation of PKA activity within the RSC.^17^ However, these molecules have a completely opposite effect on other affective processes, such as depression-related behavior, where inhibition of NR2B and enhancement of PKA mediate rapid antidepressant actions.^25, 26^ Thus, although downregulation of NR2B improves depression-related symptoms,^27, 28^ it simultaneously promotes persistent fear.^29^

To better understand the roles of NR2B and PKA in affective processes, we attempted to dissect the roles of localized NR2B and PKA signaling in behavioral phenotypes that have been associated with changes of fear, anxiety and mood. Docking of receptors and signaling molecules to scaffold proteins provides spatial, temporal and substrate specificity to glutamatergic signaling by bringing together groups of chemically different, but functionally related, bioactive molecules.^30^ As a result, knockout, knockdown or knock-in of glutamate receptor scaffolds typically affects a subset of specific behaviors, rather than causing broad behavioral deficits as do receptor agonists or antagonists.^31, 32, 33^ We focused on scaffolds regulating NR2B and PKA that include receptor for activated C kinase 1 (RACK1), which inhibits NR2B function,^34^ and A-kinase anchor protein (AKAP), and microtubule-associated protein (MAP) 2, which enhance PKA activity.^35, 36, 37^ Consistent with our hypothesis that interference with localized PKA signaling would not result in general neuronal dysfunction, knockout or mutations of AKAP5 cause neuronal redistribution of PKA regulatory subunit II (PKA-RII) and alterations of synaptic plasticity, but only slight deficits in hippocampus-dependent memories.^38, 39^ Finally, NR2B binds to MAP2 (ref. 40) and docks a substantial portion of the total neuronal PKA pool in layer 2,3 cortical neurons,^41^ in which we have observed changes of RSC PKA signaling during remote fear extinction.^17^ These complexes can be selectively disrupted by competing peptides that contain the short sequences required for specific recognition by the interacting partner. Fusion of these peptides to the transduction domain of the HIV-1 transactivating regulatory protein (Tat) makes them cell permeant and highly effective *in vivo*,^42, 43^ by blocking post-receptor PKA signaling without affecting essential receptors functions or their insertion into the neuronal membrane.^44, 45^ Uncoupling NR2B from RACK1 by Tat-RACK1 triggers localized activation of NR2B,^34^ whereas uncoupling of PKA from AKAP or MAP2 using Tat-AKAP or Tat-MAP2 causes localized downregulation of PKA.^46^ These latter peptides can thus be used to directly inhibit PKA activity localized at AKAP or MAP2 and thus circumvent the need for activation of NR2B. Here, we used them to study the roles of RACK1/NR2B, AKAP/PKA and MAP2/PKA complexes in the RSC and establish whether mechanisms mediating remote fear extinction from other anxiety- and depression-related behaviors can be differentiated at the level of discrete NR2B and PKA signaling. In addition to behavior, we also investigated whether discrete manipulation of NR2B and PKA signaling affect the expression of their downstream target brain-derived neurotrophic factor (Bdnf), which has been linked to anxiety, depression and responses to antidepressants.^47, 48, 49^ The *Bdnf* gene shows unique complexity, with only one coding exon (IX) and eight non-coding exons (I–VIII) that are believed to affect the localization and function of Bdnf.^50^ To date, abnormal expression of Bdnf exon IV transcripts has been linked to anxiety and depression,^51^ exon VI to fear extinction^52, 53^ and exon I to major depression.^54^ We therefore focused on these Bdnf transcripts.

## Materials and methods

### Subjects

Nine-week-old male C57BL/6N mice were purchased from Harlan (Indianapolis, IN, USA), individually housed (12-h light/dark cycle (lights on at 0700 hours), 40–60% humidity, 20–22 °C) and allowed *ad libitum* access to food and water. All procedures were approved by Northwestern University's Animal Care and Use Committee and are in compliance with National Institutes of Health standards.

### Surgery and cannulation

Mice were anesthetized with 1.2% tribromoethanol (Avertin), mounted in a digital stereotaxic apparatus (Kopf Instruments, Tujunga, CA, USA) and implanted with double-guide cannulas (26-gauge outer and 28-gauge inner diameter, 0.8 mm center-to-center distance; Plastics One, Roanoke, VA, USA) aimed at RSC (1.8 mm posterior, ±0.4 mm lateral, 0.75 mm ventral to bregma). The mice were allowed to recover for 1 week before injection of Tat peptides. At the end of each experiment, brains were collected for histological verification of cannula placements, unless used for messenger RNA (mRNA) preparation for analyses of Bdnf transcripts. Only data from mice with correct cannula placements were used for statistical analyses.

### Treatments and infusions

The specific NR2B antagonist Ro25-6981 was diluted in 10% dimethyl sulfoxide to a concentration of 2 μg μl^−1^ and infused into RSC (0.2 μl per hemisphere). For fear extinction, infusions were performed immediately after each of the seven daily extinction sessions. For novelty-suppressed feeding, the same dose of Ro25-6981 was infused once, 30 min before the test.

All peptides were synthesized by CHI Scientific (Maynard, MD, USA) and contained an *N*-terminal Tat sequence (YGRKKRRQRRR) conferring cell permeability and a C-terminal scaffold-specific sequence. Tat-RACK1 (YGRKKRRQRRRLYGKFSFKSDRYS) disrupts the NR2B/RACK1 interaction;^34^ Tat-AKAP (YGRKKRRQRRRQIEYVAKQIVDYAIHQA) disrupts the interaction between AKAP and PKA-RII;^55^ Tat-MAP2 (YGRKKRRQRRRDRETAEEVSARIVQVVTAEAVAVLKGEQEKE) disrupts MAP2/PKA-RII interactions.^35^ The scrambled control peptide contained the Tat sequence followed by a computer-generated scrambled sequence (YGRKKRRQRRRKFSFYVAKQRIVQVGEQEKY) containing the amino acids present in the other peptides. Tat peptides were infused bilaterally into RSC at a dose of 250 μg per 0.2 μl per site over 2 min (0.1 μl min^−1^) under a light isoflurane anesthesia immediately after each extinction test. Pilot dose–response studies established that this dose effectively disrupted NR2B or PKA interactions with RACK1, AKAP and MAP2 (Supplementary Figure 2). Each peptide was injected once per day over 7 consecutive days, as illustrated in the experimental design of each behavioral study, because 1 week was required for extinction in the control group. The same number of injections was used for all other paradigms to facilitate comparisons and interpretation.

MK-801 was injected intraperitoneally at a dose of 1 mg kg^−1^, a dose that reduces depression-like behavior without affecting locomotion.^56^

### Fear conditioning and extinction

Contextual fear conditioning was employed to model long-term stress-related episodic memory. This type of learning is processed within hippocampal–cortical circuits and requires NR2B signaling for subsequent fear extinction.^17^ Conditioning took place in a 35 × 20 × 20-cm Plexiglas chamber with a stainless steel rod floor (4 mm diameter, 0.9 cm center to center) located within a sound-attenuating cabinet with black inner walls (TSE Systems, Bad Homburg, Germany). The box was cleaned after each mouse with 70% ethanol. Mice were placed in the chamber and, 3 min later, presented with a footshock (2 s, 0.7 mA, constant current). Mice were tested for fear to the conditioning context by returning them to this chamber for a 3-min test the following day. After 35 days, the mice were given seven daily extinction trials, which consisted of exposures to the conditioning context without shock. Freezing was scored every 5 s by a trained observer blind to the experimental conditions, expressed as the percentage of total number of observations^57^ and used as an index of fear.

### Novelty-suppressed feeding

The novelty-suppressed feeding test was used to measure the effects of Tat peptides on depression-like behavior alone or in the presence of the *N*-methyl-d-aspartate receptor antagonist MK-801. This test is sensitive to the rapid antidepressant effects of MK-801 and other NMDA receptor antagonists,^56, 58^ allows for the delineation of depression-like behavior from locomotor activity or other side effects and can be performed automatically using computerized tracking systems. Mice were deprived of food overnight and water for 3 h before testing, which is sufficient to produce a state of mild hunger but little discomfort. The test apparatus was a brightly lit open field (50 × 50 cm) enclosed by 20-cm walls. A single food pellet was placed in the center of the field prior to a mouse being placed in the periphery. A video tracking system (Videomot II, TSE Systems) monitored the latency to approach the food pellet, time spent and number of visits in the pellet area (a 10-cm diameter circle in the center), and total distance traveled during the 5-min trial. Determination of the latency to start eating was made by an observer blind to the experimental conditions. Increased latencies to approach and eat the food pellet indicated decreased motivation/increased depression-like behavior, whereas a decrease of these measures after acute treatment with MK-801 indicated antidepressant effects.^56, 59^

### Forced-swim test

Depression-like behavior was also assessed using the forced-swim test, as described previously.^60^ Mice were placed in an upright cylinder (4 l, 20 cm diameter) filled with lukewarm water (26 °C) up to 10 cm below its opening. Mice were observed for 6 min, and the amount of time spent in an immobile posture during the last 5 min was scored by two observers unaware of the experimental condition. Behavior was also recorded with a camera connected to a DVD player. Data were expressed as percent time spent in floating relative to total swim time.

### Immunohistochemistry

Brains were fixed with 4% paraformaldehyde, dehydrated in 30% sucrose and stained using the Vectastain system (Vector Labs, Burlingame, CA, USA), as described previously.^57^ Immunostaining with mouse anti-NR2B (1:2000; Abcam, Cambridge, MA, USA), followed by a secondary goat anti-mouse antibody and avidin–biotin complex, was visualized with diaminobenzidine. Sections were observed with a light microscope (Leica, Buffalo Grove, IL, USA) to verify the expression of NR2B in RSC.

### Co-immunoprecipitation and immunoblot

Protein samples were prepared from the RSC collected 1 h after the last behavioral test. Membrane fractions from two different mice from the same group were pooled to obtain 175 μg per co-immunoprecipitation. Thus, from a total of six RSC per group, we created three samples per group that were further analyzed. Immunoprecipitations were performed using the Catch and Release Kit (Millipore, Billerica, MA, USA) according to the included user's manual. Complexes were isolated with 4 μg per reaction of (mouse-anti-NR2B, 1:1000, Millipore 06-600) or mouse anti-PKA-RII (1:500, BD Transduction Laboratories, San Jose, CA, USA, Clone 45 RUO) or 4 μg mouse IgG (Sigma, St. Louis, MO, USA) used as a negative control. Eluates and inputs were reduced in loading buffer with dithiothreitol and boiled for 5 min before electrophoresis. Samples were subjected to electrophoresis on 7.5% (MAP2) or 12% (RACK1 and AKAP) SDS-polyacrylamide gels and transferred to polyvinylidene fluoride membranes (Millipore). Membranes were blocked with I-Block (Life Technologies, Carlsbad, CA, USA), incubated with the following primary antibodies: rabbit-anti-RACK1 (Abcam, ab129084), mouse anti-anti-MAP2 (Abcam, ab 11267), rabbit anti-AKAP150 (Santa Cruz, Dallas, TX, USA, C-20), anti-NR2B, anti-PKA or anti-β-tubulin (Sigma, T8328) overnight at 4 °C, followed by corresponding secondary antibody (Goat Anti-Rabbit 1:10 000; Santa Cruz) for 4 h at room temperature. Membranes were then incubated with alkaline phosphatase chemiluminescence enhancer (Nitro-Block II, Life Technologies) and substrate CDP Star (Life Technologies), and then exposed to X-ray film for detection. Quantification was performed densitometrically using ImageJ software (NIH, Bethesda, MD, USA). Immunoblot analyses of pro-Bdnf and Bdnf were performed similarly, using anti-Bdnf (Millipore, AB1779). For co-immunoprecipitation, data were normalized to the amount of primary precipitated proteins (NR2B or PKA), whereas for pro-Bdnf and Bdnf, data were normalized to β-tubulin.

### Real-time PCR for Bdnf transcripts

Brains were dissected and RSC was collected. Tissue was homogenized in lysis buffer with β-mercaptoethanol and frozen in liquid nitrogen. RNA was extracted using the miRCURY total RNA isolation kit (Exiqon, Woburn, MA, USA), reversely transcribed and subjected to real-time polymerase chain reaction (PCR) using the SYBR Green master mix (Life Technologies) and primers for Bdnf transcripts containing exons I–IX or mouse cyclophillin as an internal control. The primers sequences were: BDNF E9 (F): 5′-GCGCCCATGAAAGAAGTAAA-3′, BDNF E9 (R): 5′-TCGTCAGACCTCTCGAACCT-3; BDNF E1 (F): 5′-CCTGCATCTGTTGGGGAGAC-3′, BDNF E1 (R): 5′-GCCTTGTCCGTGGACGTTTA-3′; BDNF E4 (F): 5′-CAGAGCAGCTGCCTTGATGTT-3′, BDNF E4 (R): 5′-GCCTTGTCCGTGGACGTTTA-3′; BDNF E6 (F): 5′-CTGGGAGGCTTTGATGAGAC-3′ BDNF E6 (R): 5′-GCCTTCATGCAACCGAAGTA-3′ cyclophilin (F): 5′-CCCACCGTGTTCTTCGACA-3′, cyclophilin (R): 5′-CCAGTGCTCAGAGCTCGAAA-3′. The PCR conditions were 95 °C for 10 min, followed by 40 cycles of 95 °C for 15 s and 60 °C for 1 min. The samples were run in triplicate and the delta-delta-CT method was used for quantification. Results for all samples were normalized to cyclophillin and expressed as fold difference from the naive group.

### Statistical analysis

We used 7–10 mice per experimental group for the behavioral studies. For quantitative PCR and immunoblot assays, we used 3–5 samples per group. These sample sizes gave us statistical power of 80% to detect treatment effects. All data showed normal distribution and were analyzed by two-tailed Student's *t*-test for two groups or one-way analysis of variance (ANOVA) for three or more groups with treatment as a main factor. Fear extinction was analyzed by repeated measure ANOVA with treatment as a factor. *Post hoc* comparisons were performed using Tukey's test. Data are presented as mean±s.e.m.

## Results

### Opposite effects of NR2B antagonism in RSC on remote fear extinction and depression-like behavior

**Figure 1:**
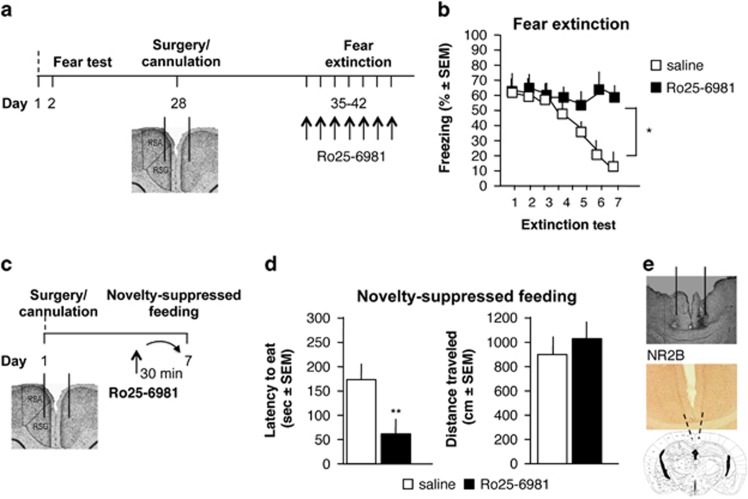
Role of RSC NR2B in fear extinction and novelty-suppressed feeding. (**a**) Procedure and treatment for remote fear extinction. (**b**) Effect of the specific NR2B antagonist Ro25-6981 on extinction of remote fear. (**c**) Procedure and treatment for the novelty-suppressed feeding test. (**d**) Effect of Ro25-6981 on the latency to eat (left) and distance traveled (right) in the novelty-suppressed feeding test. (**e**) Photomicrograph of a representative cannula placement (top), and NR2B-positive neurons (middle) in the targeted RSC area (bottom). *P<0.05, **P<0.01 vs vehicle. RSC, retrosplenial cortex.

To establish whether NR2B within the RSC regulates both remote fear extinction and depression-related behavior, we first infused the NR2B antagonist Ro25-6981 or vehicle into RSC after each extinction trial, starting 35 days post training (Figure 1a). The controls showed significant reduction of remotely acquired fear after repeated exposures to the context without shock, whereas infusions of Ro25-6981 resulted in persistent freezing (*n*=8–9 per group; main effects of drug: F(1,15)=9.15, *P<0.01, day: F(6,90)=19.37, *P*<0.001 and drug × day interaction F(6,90)=8.77, *P*<0.001 vs saline; Figure 1b). This effect of the NR2B antagonist was relatively specific for remote fear extinction, as revealed by the findings that extinction of recently acquired fear and retrieval of recent or remote memory were intact (Supplementary Figure 1).

A single infusion of Ro25-6981 before the novelty-suppressed feeding test (Figure 1c) significantly reduced the latency to eat (*n*=10 per group; *t*18=6.55, **P<0.01) (Figure 1d, left) without affecting distance traveled (*t*18=1.12, *P*=0.14) (Figure 1d, right) when compared with the saline controls. Immunohistochemical analysis demonstrated NR2B-positive cells at the neuroanatomical level of RSC used for cannulation and treatment (Figure 1e). These findings demonstrate that antagonism of RSC NR2B abolishes remote fear extinction while exerting a rapid antidepressant action, and implicate RSC as a brain area in which fear and mood are counter-regulated.

### NR2B and PKA signaling complexes in the RSC are disrupted by Tat peptides

**Figure 2:**
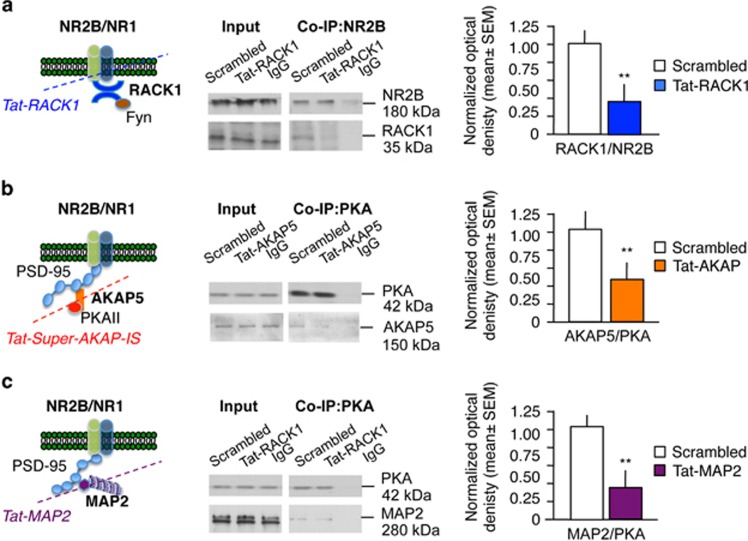
Disruption of RACK1, AKAP and MAP2 complexes after *in vivo* intra-RSC infusions of Tat peptides. (**a**) NRB/RACK1 interaction after Tat-RACK1 infusions. (**b**) PKA/AKAP5 interaction after Tat-AKAP infusions. (**c**) PKA/MAP2 interaction after Tat-MAP2 infusions. **P<0.01 vs scrambled peptide. AKAP, A-kinase anchor protein; MAP, microtubule-associated protein; PKA, protein kinase; RACK1, receptor for activated C kinase 1; RSC, retrosplenial cortex.

The effects of Tat peptides on interactions between scaffolding proteins and NR2B or PKA were established by co-immunoprecipitation of these proteins in RSC lysates obtained 1 h after the last extinction test. On the basis of pilot data (Supplementary Figure 2), 0.5 μg per 0.4 μl of each peptide were infused 0.2 μl per site over 7 days immediately after each extinction test. Infusions of Tat-RACK1 significantly reduced the binding of NR2B to RACK1 (*n*=6 per group; *t*11=8.23, **P<0.01; Figure 2a), whereas Tat-AKAP and Tat-MAP2 significantly reduced the binding of PKA to AKAP and MAP2 (AKAP: *n*=6 per group; *t*11=6.45, **P<0.01; MAP2: *n*=6 per group; *t*11=6.98, **P<0.01) when compared with the scrambled peptides; Figures 2b and c), respectively. Thus, the Tat peptides effectively disrupted the specific protein–protein interactions, which were reduced by 60–75%.

### Effects of intra-RSC infusions of Tat-RACK1, Tat-AKAP and TAT-MAP2 on remote fear extinction

**Figure 3:**
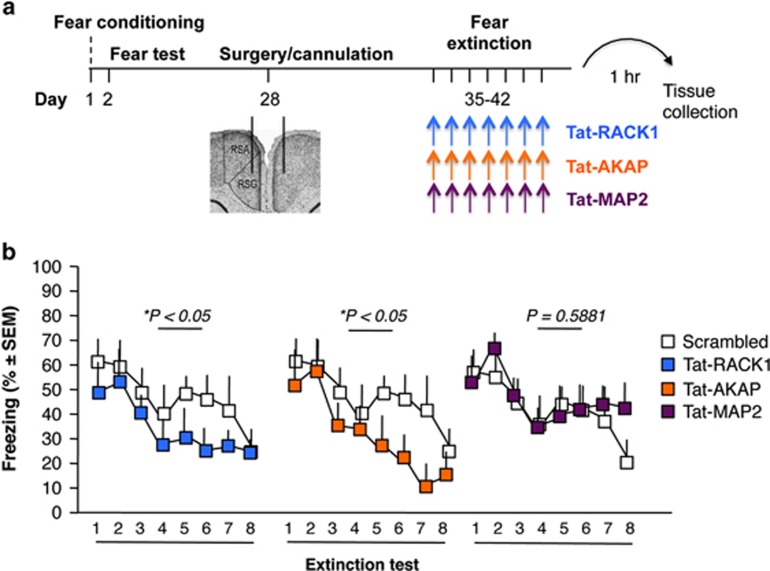
Effects of disrupting NR2B/RACK1, AKAP/PKA or MAP2/PKA complexes in RSC on extinction of remotely acquired fear. (**a**) Experimental design and treatment schedule. (**b**) Effects of post-test RSC infusions of Tat-RACK1, Tat-AKAP and Tat-MAP2 on freezing behavior. *P<0.05 vs scrambled peptide. AKAP, A-kinase anchor protein; MAP, microtubule-associated protein; PKA, protein kinase; RACK1, receptor for activated C kinase 1; RSC, retrosplenial cortex.

Daily post-test intra-RSC infusions (Figure 3a) of Tat-RACK1 and Tat-AKAP peptides significantly enhanced fear extinction, whereas Tat-MAP2 was ineffective (Figure 3b). Two-factor ANOVA, with factors treatment and test, identified significant differences for Tat-RACK1 (*n*=8–9 mice per group; F(1,7)=4.776, *P*<0.05) and Tat-AKAP (*n*=8–9 mice per group; F(1,7)=8.73, *P*<0.05), but not Tat-MAP2 (*n*=8–9 mice per group; F(1,7)=0.02, *P*=0.962). These findings showed that extinction of remotely acquired fear can be enhanced by interference with discrete NR2B or PKA complexes.

### Effects of intra-RSC infusions of Tat-RACK1, Tat-AKAP and TAT-MAP2 on anxiety- and depression-related behavior

**Figure 4:**
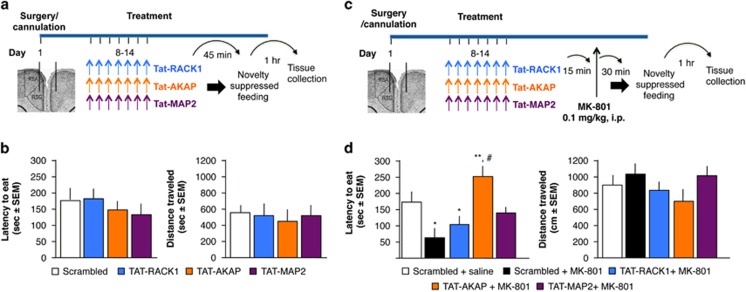
Effect of disrupting NR2B/RACK1, AKAP/PKA or MAP2/PKA complexes in RSC on depression-related behavior. (**a**) Experimental design and treatment schedule for the novelty-suppressed feeding test. (**b**) Effects of post-test RSC infusions of Tat-RACK1, Tat-AKAP and Tat-MAP2 latency to eat (left) and total distance traveled (right). (**c**) Experimental design for the antidepressant response to MK-801. (**d**) Effects of of Tat-Rack1, Tat-AKAP and Tat-MAP2 on the latency to eat (left) and distance traveled (right) in the novelty-suppressed feeding test following injection of MK-801. *P<0.05, **P<0.01 vs Scrambled+saline control; ^#^*P*<0.05 vs Scrambled+MK-801 group. AKAP, A-kinase anchor protein; MAP, microtubule-associated protein; PKA, protein kinase; RACK1, receptor for activated C kinase 1; RSC, retrosplenial cortex.

To produce a similar biochemical effect on NR2B and PKA complexes as in the previous experiment, mice were given seven daily intra-RSC infusion of Tat-RACK1, Tat-AKAP or Tat-MAP2. The novelty-suppressed feeding test was performed after the seventh infusion (Figure 4a). None of the Tat peptides affected the latency to eat the food pellet or locomotor activity (*n*=7–8 mice per group; latency to eat: F(3,26)=1.344, *P*=0.284; locomotor activity: F(3,26)=0.843, *P*=0.484; Figure 4b). The peptides were similarly ineffective in modulating the time spent in the center of the open field during the novelty-suppressed feeding test (F(3,26)=0.576, *P*=0.45) or floating time in the forced-swim test (F(3,26)=1.721, *P*=0.188) (Supplementary Figure 3). Thus, despite the finding that some treatments enhanced fear extinction, none of the Tat peptides alone had any effect on anxiety- or depression-like behavior in general.

In separate groups of similarly treated mice, we determined whether disruption of NR2B/RACK1, PKA/AKAP or PKA/MAP2 affects the rapid antidepressant action of the glutamatergic antagonist MK-801 (Figure 4c). At 15 min after the last Tat peptide infusion, mice were injected intraperitoneally with 0.1 mg kg^−1^ of MK-801 and tested 30 min later. One-way ANOVA identified significant group differences, using treatment as a main factor, for latency to eat the food pellet (8 mice per group; F(4,35)=3.180, *P*<0.05) without significantly affecting locomotor activity (F(4,35)=1.82, *P*=0.19). *Post hoc* analyses showed that, consistent with earlier observations,^56^ MK-801 significantly reduced the latency to eat the food pellet (*P*<0.01; Figure 4d) in mice pre-treated with Tat-scrambled peptide (Scrambled+MK-801) as compared with the control treatment (Scrambled+saline). Tat-RACK-1 did not alter the effect of MK-801, as mice in this group behaved similarly as mice in the Scrambled+MK-801 group. Tat-MAP2 blocked the effect of MK-801, as revealed by lack of significant MK-801 effects in this group on latency to eat (*P*=0.238). However, Tat-AKAP not only blocked the anxiolytic and antidepressant actions of MK-801, but it inverted them into depression-enhancing actions, as revealed by significantly increased latency to eat (*P*<0.01) when compared with both the Scrambled+saline and Scrambled+MK-801 groups. Similarly, Tat-AKAP reduced the time spent in the center of the open field (F(4,32)=3.25, *P*<0.05) and increased the time spent floating in the forced-swim test (F(4,32)=7.35. *P*<0.001) (Supplementary Figure 3).

Together these findings demonstrate that the Tat peptides did not induce anxiety or depression-like behavior in the novelty-suppressed feeding and forced-swim tests; however, anxiolytic and antidepressant responses were markedly influenced by disruption of PKA complexes, in particular PKA/AKAP, but not the RACK1/NR2B complex.

### Effects of Tat-RACK1, Tat-AKAP and TAT-MAP2 infused into the RSC on the expression of Bdnf transcripts

**Figure 5:**
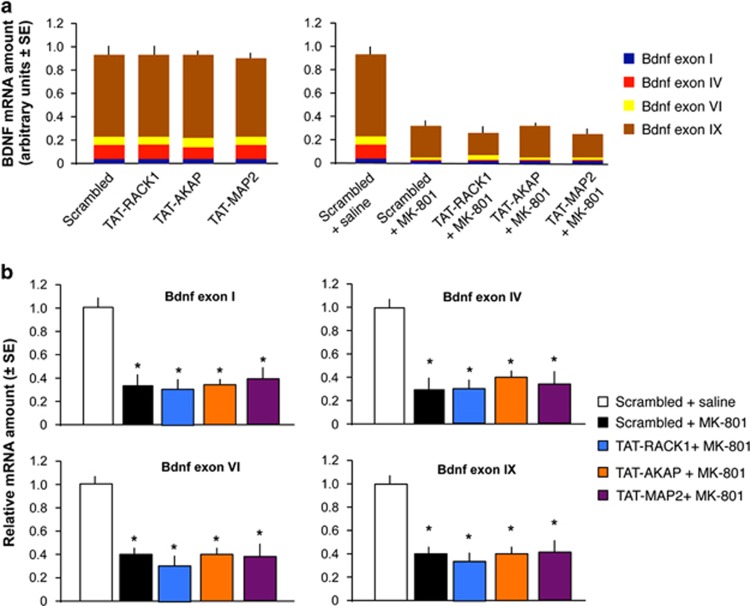
Effect of disrupting NR2B/RACK1, AKAP/PKA or MAP2/PKA complexes on the levels of BDNF transcripts. (**a**) Tat peptides administered without (left) or with (right) MK-801 treatment did not affect the expression of Bdnf transcripts. (**b**) Comparisons of normalized levels of the individual transcripts showed in **a**. *P<0.05 vs Scrambled+saline control. AKAP, A-kinase anchor protein; BDNF, brain-derived neurotrophic factor; MAP, microtubule-associated protein; mRNA, messenger RNA; PKA, protein kinase A; RACK1, receptor for activated C kinase 1.

The seven daily infusions of Tat peptides did not affect the expression of Bdnf mRNA transcripts containing exons I, IV, VI or IX (F(4,8)=7.85, *P*<0.01; Figure 5a). Acute injection of MK-801 resulted in a significant reduction of all of these Bdnf transcripts, but this effect was not influenced by any of the Tat peptides (Figure 5b). One-way ANOVA revealed significant effects of treatment for Bdnf exon I (F(5,10)=8.17, *P*<0.01), exon IV (F(5,10)=9.32, *P*<0.01), exon VI (F(5,10)=9.27, *P*<0.01) and exon IX (F(5,10)=8.94, *P*<0.01) transcripts. *Post hoc* analyses showed that all MK-801-treated mice had similar decreases of Bdnf mRNA levels independent of the Tat peptide treatment. To establish whether the observed changes might be due to enhanced translation of Bdnf, we also determined the levels of Bdnf and its precursor pro-Bdnf. We found a small but significant increase of the level of both polypeptides (pro-Bdnf: F(4,15)=4.973, *P*<0.01; Bdnf: F(4,15)=4.204, *P*<0.05; Supplementary Figure 4). This suggests that increased translation might contribute to the decreased levels of Bdnf transcripts, however, given the sizes of the observed effects on Bdnf mRNA versus protein levels, MK-801 could also have disrupted transcription.

## Discussion

Here we demonstrated that disruption of discrete NR2B and PKA complexes within RSC significantly affects extinction of remotely acquired fear and affective behavior. Whereas the NR2B/RACK1 complex proved specific for fear extinction and PKA/MAP2 for antidepressant and anxiolytic responses, PKA/AKAP was identified as the pathway affecting both behaviors but in opposite directions. These findings suggest that interference with specific signaling complexes, rather than general receptor or PKA populations, has the potential to improve fear extinction deficits without adversely affecting mood and motivation and vice versa.

Substantial advances have been made in our understanding of fear triggered by recent stressful experiences,^61, 62^ but it is not well understood how the passage of time affects fear regulation. It is increasingly recognized, based on phenomenological, circuit and molecular evidence, that extinction of recently and remotely acquired fear requires different mechanisms.^17, 63, 64, 65^ Increased NR2B and suppressed PKA activity in different regions of the fear circuit, such as hippocampus, amygdala and prefrontal cortex, are required for recent fear extinction;^21, 66, 67^ however, as memory ages, these mechanisms increasingly involve RSC.^17^ Therefore, the roles of RACK1 and AKAP complexes in the RSC identified in this study may be relevant for extinction of both recently and remotely acquired fear.

Although activation of NR2B seems like an obvious approach for facilitating fear extinction and reducing fear symptoms, this is complicated by the facts that NR2B-specific agonists are not available and would likely exert adverse effects on mood. We successfully overcame both limitations by using the Tat-RACK1 peptide, which by disrupting NR2B/RACK1 interactions, enhances the phosphorylation and activity of NR2B.^34^ The finding that Tat-RACK1 enhanced extinction without affecting depression-related behavior also showed that discrete NR2B/RACK1-mediated mechanisms contribute to fear versus mood regulation. Tat-AKAP enhanced fear extinction similar to Tat-RACK1, suggesting that decreased PKA activity within a specific scaffolding/signaling complex might suffice to reduce fear even without NR2B activation.

Interference with RACK1, AKAP or MAP2 complexes did not exert notable effects on baseline levels of mood and motivation, as shown by lack of effects on the latency to eat in the novelty-suppressed feeding test, suggesting that their effects might be specific for fear extinction. However, this is unlikely, given the significant involvement of the PKA-associated scaffolds in the antidepressant response, and requires further research using stress- or genetically induced depression-like behavior. Antidepressant responses have increasingly been used as indicators of depression-related behavior, because they are robust, reproducible and occur in response to drugs that target neurotransmitter systems relevant for human symptoms.^58, 68, 69^ This approach proved more sensitive than the use of behavioral paradigms alone in identifying a role of the serotonin Htr4 receptor scaffold p11 in depression-related behavior,^70^ and we applied it here to study antidepressant responses to glutamatergic signaling. Among rapidly acting glutamatergic antidepressants, ketamine has been investigated the most;^68^ however, we focused on MK-801 because its behavioral and molecular effects are particularly robust,^56^ which facilitates studying molecular mechanisms and modulatory effects. Antidepressant actions of MK-801 significantly depend on AKAP- and MAP2-associated signaling, but do not involve RACK1/NR2B. This finding confirms the role of PKA activity in depression-related behavior,^71^ and further demonstrates that distinct subcellular pools of PKA differentially affect the antidepressant effect of MK-801. Namely, whereas intact MAP2/PKA was required for the MK-801 response in general, AKAP/PKA was critical for the direction of MK-801's effect. That disrupting AKAP/PKA complexes MK-801 induced depression-like behavior instead of an antidepressant effect was perhaps the most striking observation in our study, and suggests that MK-801 triggers both pro- and antidepressant signaling pathways. MAP2/PKA seems to be required for both, because when this complex was disrupted, MK-801 was completely ineffective. However, when AKAP/PKA was disrupted, signaling most likely proceeded via pathways mediating depression-related behavior, resulting in corresponding changes of motivation in the novelty-suppressed feeding test. Given that we used a Tat-AKAP peptide that specifically disrupts the interaction of AKAP with PKA RII,^55^ thus this PKA isoform is likely the mediator of the antidepressant effect of MK-801. Interestingly, another AKAP-interacting signaling molecule, the protein phosphatase calcineurin,^72^ mediates the antidepressant effects of the serotonin reuptake inhibitor fluoxetine^73^ and serotonin and norepinephrine reuptake inhibitor venlafaxine.^74^ The multiprotein complexes scaffolded by AKAP might thus emerge as key mediators of depression-related behaviors and potential novel targets for antidepressant treatments.

MK-801 induced significant decreases in the levels of Bdnf mRNA transcripts, including the transcripts coding for exons I, IV, VI and IX. This finding was unexpected in light of earlier reports that the low dose of MK-801 used enhances Bdnf protein synthesis but not *Bdnf* transcription 30 min after MK-801 injection.^56^ Because our samples were collected 1 h later, increased translation into Bdnf protein most likely depleted Bdnf mRNA levels, resulting in a decrease of all Bdnf transcripts. Alternatively, MK-801 may have inhibited *Bdnf* transcription as an independent later effect. If so, such effect would be an opposite from actions of high doses of MK-801 that are known to enhance Bdnf transcription in RSC.^75, 76^ Treatment with Tat peptides did not affect the levels of any of the major Bdnf transcripts in RSC, suggesting that discrete manipulations of NR2B and PKA complexes, unlike broad NR2B and PKA activators or inhibitors, do not interfere with Bdnf gene expression. This result is surprising, given the profound differences in behavioral responses to the three Tat peptides. Thus, at least some fear- and depression-related behaviors related to rapid antidepressant or anxiolytic effects can be modulated by NR2B- and PKA-mediated mechanisms independently of changes in Bdnf levels. It remains to be established whether such Bdnf-independent mechanisms operate solely within the RSC or also in other brain areas.

In summary, our findings demonstrate that the subcellular organization of NR2B and PKA signaling complexes within RSC is a major determinant of their roles in remote fear extinction and depression-related behavior. Therefore, these complexes are potential therapeutic targets for fear and mood symptoms in patients with comorbid PTSD and depression. With innovative application strategies that improve peptide bioavailability, in particular the use of nanotechnology, cell-permeant, complex-specific peptides could be a promising approach as patients could benefit from their high efficacy, high specificity and low toxicity.^76^
