# Supplementary figures and images for: Regulation of fear extinction versus other affective behaviors by discrete cortical scaffolding complexes associated with NR2B and PKA signaling
Source: Transl Psychiatry. 2015 Oct 13;5(10):e657–. doi: 10.1038/tp.2015.150 (PMC4930127; doi:10.1038/tp.2015.150)

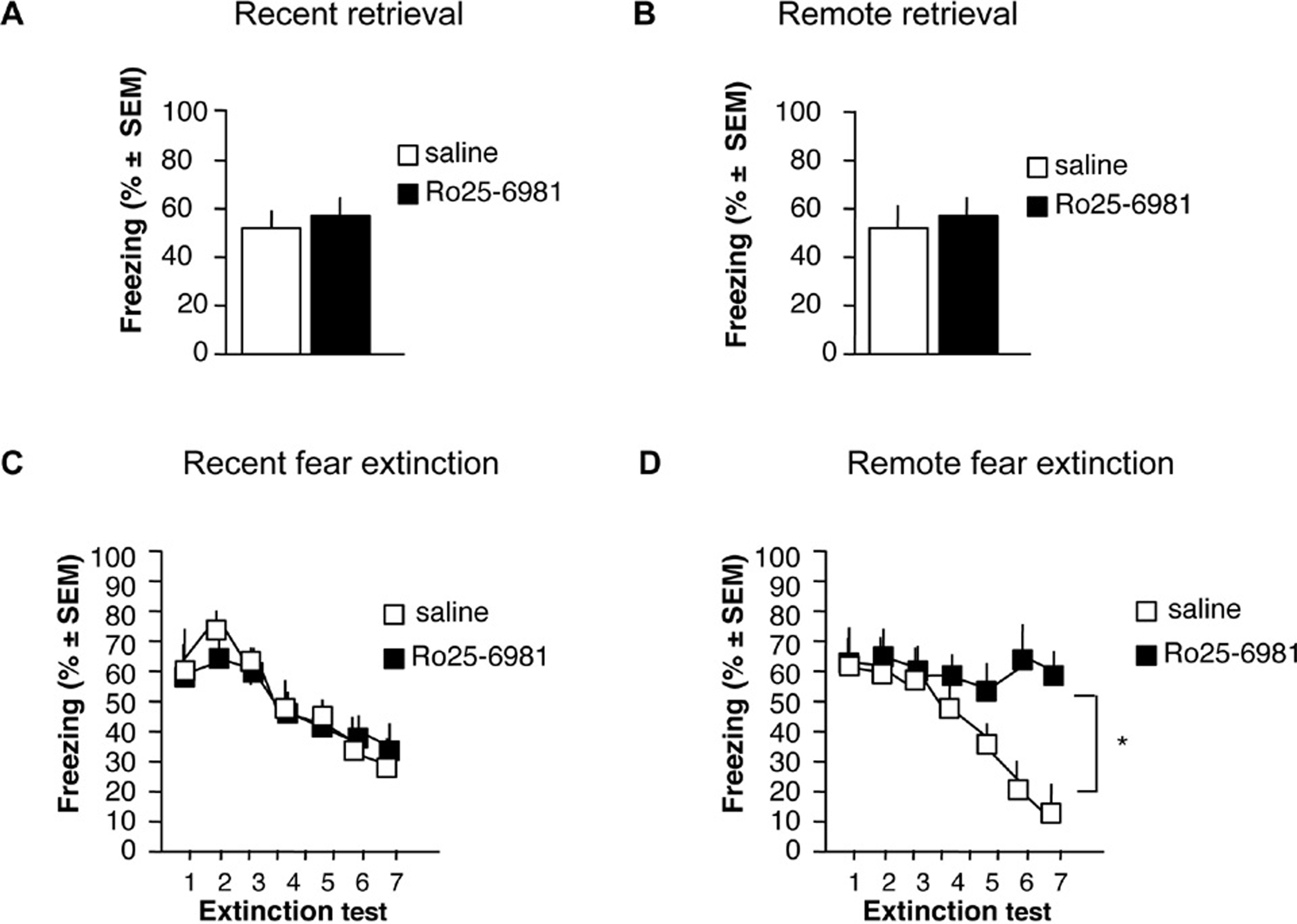

Supplement: Supplementary Figure 1 [file tp2015150x1.tif]

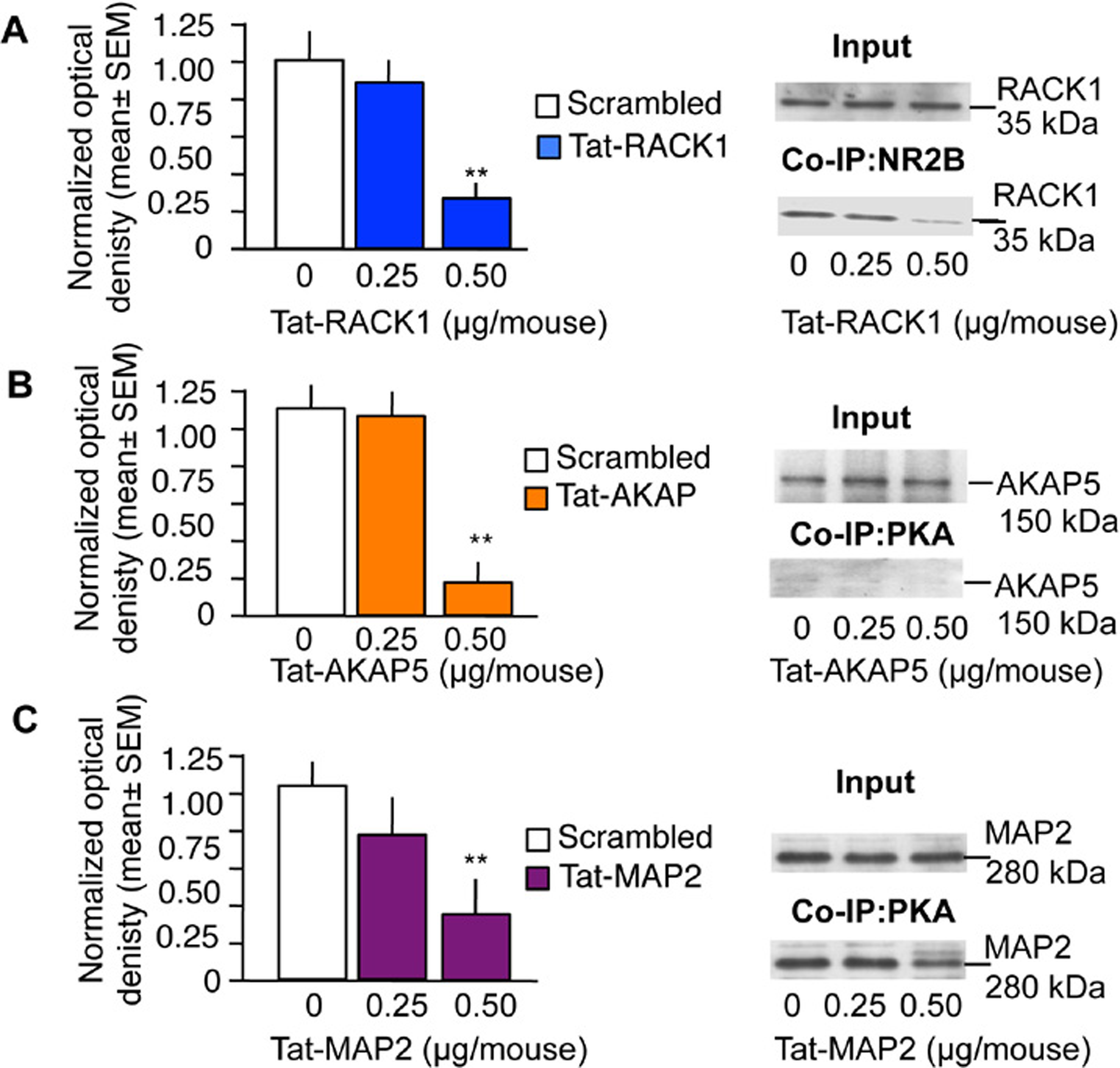

Supplement: Supplementary Figure 2 [file tp2015150x2.tif]

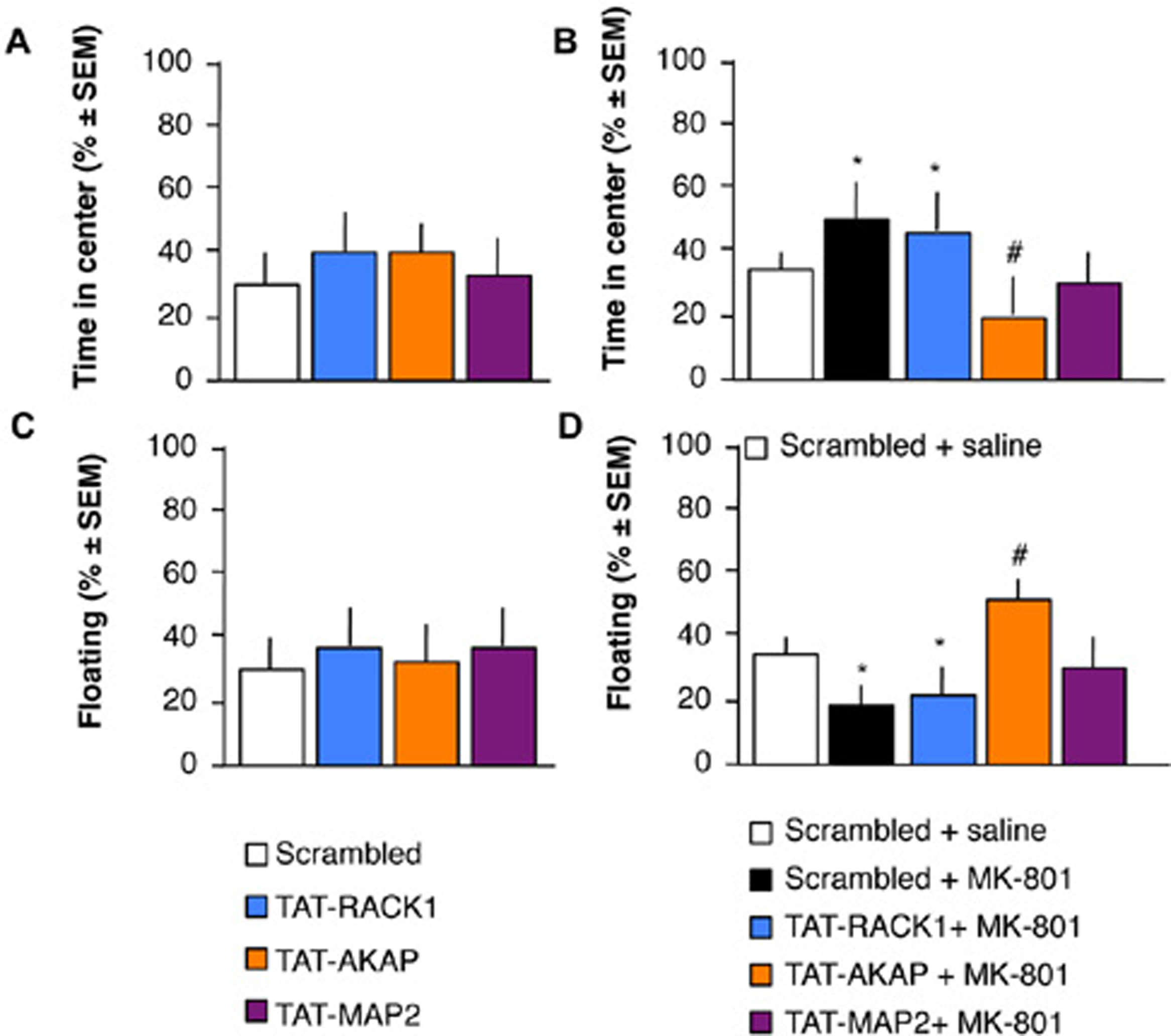

Supplement: Supplementary Figure 3 [file tp2015150x3.tif]

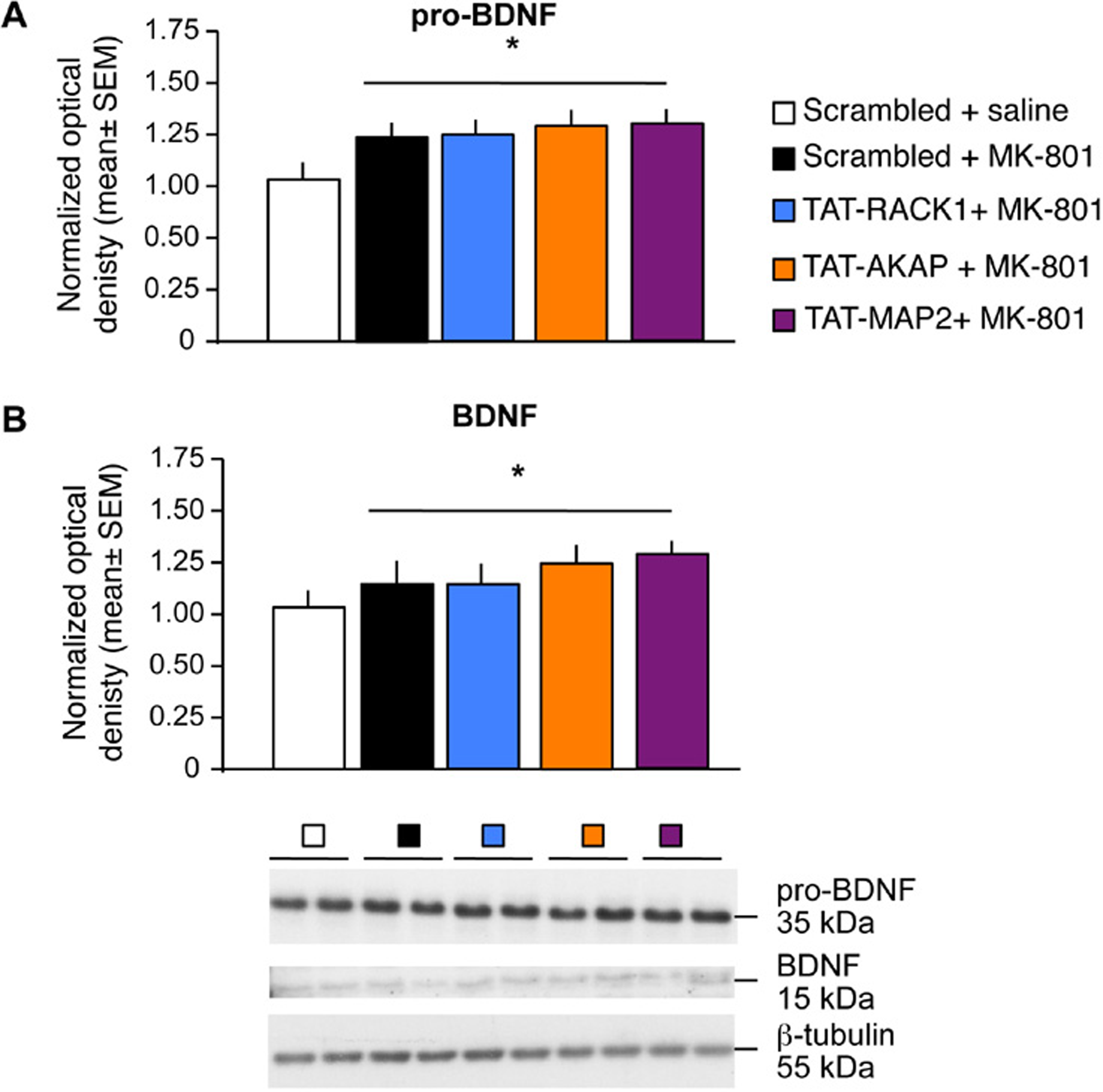

Supplement: Supplementary Figure 4 [file tp2015150x4.tif]
