# Supplementary material for: Regulation of fear extinction versus other affective behaviors by discrete cortical scaffolding complexes associated with NR2B and PKA signaling
Source: Transl Psychiatry. 2015 Oct 13;5(10):e657–. doi: 10.1038/tp.2015.150 (PMC4930127; doi:10.1038/tp.2015.150)
Supplement: Supplementary Figure Legends [file tp2015150x5.docx]

**LEGENDS FOR SUPPLEMENTARY FIGURES**

**Figure 1.** Role of RSC NR2B in memory retrieval and extinction of recently and remotely acquired fear-provoking memories. (**a**) The specific NR2B antagonist Ro25-6981 did not affect retrieval of recently acquired fear (n = 7 mice/group; t1,13 = 0.1; *P* = 0.92) after RSC infusion as determined by Student’s t-test. (**b**) Ro25-6981 did not affect retrieval of remotely acquired fear (n = 7 mice/group; t1,13 = 0.16; *P* = 0.7). (**c**) Ro25-6981 did not affect extinction of recently acquired fear, as revealed by lack of treatment effects (n = 8-9 mice/group; F1,13 = 0.32; *P* = 0.58) or treatment x test interaction (F1,78 = 0.86; *P* = 0.53) using two-way ANOVA. (**e**) In contrast, Ro25-6981 abolished extinction of remotely acquired fear, resulting in significant effects of treatment (F1,15 = 9.15; *P* < 0.01) and treatment x test interactions (n = 7 mice/group; F1,90 = 9.15, P < 0.001). **P* < 0.05, ***P* < 0.01 vs vehicle.

**Figure 2.** Disruption of RACK1, AKAP, and MAP2 complexes after *in vivo* intra-RSC infusions of Tat peptides. This was a pilot study with 3 mice/group, and data were normalized to the input proteins. (**a**) NRB/RACK1 interaction after Tat-RACK1 infusions ( F2,6 = 4.91, P < 0.01); (**b**) PKA/AKAP5 interaction after Tat-AKAP infusions (F2,6 = 5.27, P < 0.001); (**c**) PKA/MAP2 interaction after Tat-MAP2 infusions MAP2 F2,6 = 4.14, P < 0.01. ***P* < 0.01 vs control without peptide.

**Figure 3.** Effects of disrupting NR2B/RACK1, AKAP/PKA, or MAP2/PKA complexes in RSC on anxiety- and depression-like behavior and responses to MK-801. (**a**) Tat peptides did not affect the time in the center of the open field during the NSF test. (**b**) Tat peptides modulated the anxiolytic effect of MK-801 on time spent in the center. (**c**) Tat peptides did not affect the floating time in the forced swim test test. (**d**) Tat peptides modulated the antidepressant effect of MK-801 on time spent floating. **P* < 0.05 vs scrambled + saline group; #*P* < 0.05 vs srambled+MK-801 group .

**Figure 4.** Effect of disrupting NR2B/RACK1, AKAP/PKA, or MAP2/PKA complexes in RSC on the changes of pro-Bdnf and Bdnf induced by MK-801. (**a**) MK-801 significantly increased the level of pro-Bdnf, but there was no effect of peptide treatments; (**b**) MK-801 significantly increased the level of Bdnf, but there was no effect of peptide treatments. **P* < 0.05, ***P* < 0.01 vs Scrambled + saline control.
